# APLNR reduction in kidney-muscle crosstalk in renal model recovered by exercise and STAT3 inhibition

**DOI:** 10.1016/j.bbrep.2026.102565

**Published:** 2026-04-13

**Authors:** Gabriel Pereira, Thabata Caroline de Oliveira Santos, Sofía Tomaselli Arioni, Pietra Mancini Seibt, Emily Pereira dos Santos, Luana Fortuna, Rodrigo Lazzarotto, Matheus Felipe Zazula, Juan Sebastian Henao Agudelo, Débora Tavares de Resende e Silva, Elizabeth Cristina Perez Hurtado, Katya Naliwaiko, Ricardo Fernandez, Danilo Cândido de Almeida, Rafael Luiz Pereira

**Affiliations:** a Federal University of Paraná, Department of Physiology, Curitiba, State of Paraná, Brazil; b Federal University of Paraná, Department of Cellular Biology, Curitiba, State of Paraná, Brazil; c Federal University of São Paulo, Nephrology Division, São Paulo, Brazil; d Central Unit of Valle del Cauca, Faculty of Health Sciences, Valle del Cauca, Tuluá, Colombia; e Federal University of Fronteira Sul, Campus Chapecó, Chapecó, Santa Catarina, Brazil; f Universidade Paulista, Programa de Pós-Graduação em Patologia Ambiental e Experimental, São Paulo, Brazil

**Keywords:** Resistance training, STAT3, Apelin receptor (APLNR), Doxorubicin, Muscle loss, Bioinformatics

## Abstract

Current evidence show that exercise has beneficial effects on skeletal muscle function in individuals with chronic kidney disease (CKD). The STAT3 signaling pathway and the apelin axis (apelin receptor, APLNR- and ligands, -apelin and/or -elabela), participate in the processes of kidney inflammation and fibrosis and both may be involved in muscle wasting during CKD. Herein we report that STAT3 pathway and APLNR are in fact involved in the muscle impairment concomitant to CKD in experimental model and in adult individuals with CKD. Male BALB/c mice were first submitted to an 8-week ladder climbing resistance training (RT) protocol and further were submitted to doxorubicin-induced experimental CKD with or without use of the STAT3 inhibitor (Stattic). The GSE157712 dataset was used to assess the muscle transcriptome profile of CKD patients. Bioinformatics’ analysis of gene set enrichment analysis (GSEA) and gene ontology (GO) were performed and the APLNR was found to be a central component of muscle response over kidney stress. Mice from the RT protocol showed a protective effect of blocking STAT3 against kidney and muscle injury markers, while both CKD individuals and CKD mice reported alterations in the APLNR muscle expression. In conclusion, the muscle tissue function is affected during CKD, which can be attenuated by a protective and synergistic effect of exercise and STAT3 inhibition. Thus, APLNR appears as a key gene associated with muscle dysfunction in CKD patients and its muscle expression can be regulated by resistance exercise.

## Highlights

• Skeletal muscle function and integrity decline during kidney disease.
• The synergism among blocking STAT3 signaling and resistance training protect mice muscle and kidney from injury.
• Apelin receptor is involved in human and mice muscle lesion during kidney injury.
• Blocking STAT3 signaling and performing exercise improve apelin receptor expression levels in the mice muscle.

## Graphical abstract

**Figure:**
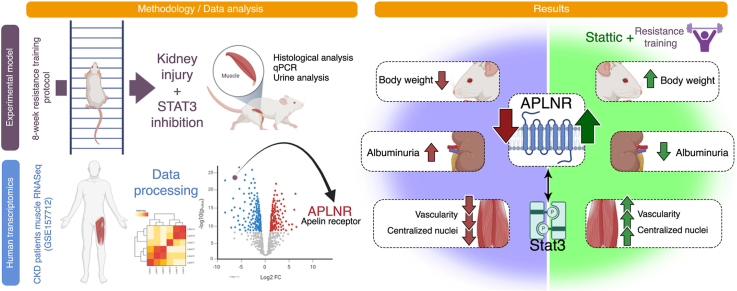

## 1 Introduction

Chronic kidney disease (CKD) is a severe worldwide health problem with an increasing prevalence, incidence, and death rate through the years. One of its prevalent complications is the associated muscle wasting during CKD, with a global prevalence of sarcopenia – decrease in muscle mass, strength and performance – of 25% of CKD patients [1].

Resistance training (RT) has been suggested to attenuate CKD markers of muscle wasting, such as creatinine kinase and systemic inflammatory response [2], and reduce the risk of death in end-stage renal disease (ESRD) patients [3]. It is known that injured kidneys release uremic toxins, pro-inflammatory cytokines, such as IL-6 and TNF-ɑ, and molecules such as activin A which act as systemic mediators of muscle wasting [4,5]. Furthermore, reduced physical performance in CKD patients and loss of muscle strength occurs often before of reduction in muscle mass during kidney disease progression [6]. Hence, the precise understanding of the muscle cell pathophysiology in CKD patients is of great importance.

Signal transducer and activator of transcription 3 (STAT3), mainly activated by interleukin (IL)-6, regulates several downstream targets of renal fibrosis-associated genes (i.e. collagen IV, TGF-β1 and VEGF). In the context of kidney injury, the STAT3 inhibition promotes reduced fibrosis and leads to low-levels of protein in the urine [[7], [8], [9]]. Moreover, STAT3 also plays a role in muscle physiology; while its activation is essential for the adaptive responses of exercise, chronic IL-6 elevation and continuously STAT3 phosphorylation are involved in activation of proteolytic pathways of muscle wasting [10,11]. The levels of phosphorylated STAT3 are higher in the muscle of CKD patients [12] and its inactivation in experimental models of muscle wasting, including CKD, have preserved muscle mass and grip strength [12,13].

Thus, the understanding of the mechanisms by which STAT3 regulate pathophysiological processes is of remarkably importance. In 2008, Han et al. found an interaction between IL-6 and STAT3 activity to a direct promoter site of the gene Apelin (APLN) in macrophages. This molecular cross-talk increase the APLN expression triggered by STAT3 activation, which led to the postulation that APLN would play a critical role in enteric inflammation [14]. Further studies on the APLN system revealed its role and interaction with STAT3 in cardiac tissue [15] along with physical exercise [16], retinal fibrosis [17] and ovarian cancer cells [18].

The Apelin receptor (APLNR, previously called APJ) have two endogenous ligands, APLN and Elabela/Toddler (ELA). The expression of the receptor and its ligands are reported in the skeletal muscle and kidney tissues [19]. In the muscle, APLNR is expressed in mature myofibers, where it regulates metabolism and hypertrophy [20]. In the kidney, the activation of APLNR is credited to be an antifibrotic agent [21,22]. Furthermore, APLN and APLNR expression in muscle decrease with aging and are associated with sarcopenia, where APLN/APLNR axis down-regulation accelerate muscle loss [20]. Interestingly it was reported that APLN supplementation can attenuate skeletal muscle atrophy in CKD mice [23].

Therefore, in attempt to elucidate the molecular mechanism behind muscle-kidney communications, the experimental design of this study was separate in two parts: i) Mice from experimental model with Doxorubicin (DOX)-induced Focal Segmental Glomerular Sclerosis (FSGS) [24] aimed to evaluate that RT improves muscle function via STAT3 signaling and, ii) bioinformatics analysis of human dataset of chronic kidney disease patients where it was examined the expression profiles of the muscle of CKD patients and healthy individuals from the dataset GSE157712 assigned at GEO database. This approach allowed us to identify potential candidate genes in regulated pathways that may play a special role in the muscle tissue function of CKD individuals. The computational analysis revealed the APLN/APLNR axis as a main signal altered in the muscle of the individuals with CKD. Further, the contribution of APLN/APLNR axis in muscle alterations was validated in experimental mice model of FSGS under exercise and STAT3 blocking. Finally, the APLNR appeared as a key gene associated to vascular and differentiation processes in the human muscle and its expression correlated to histomorphological changes in the mice muscle, suggesting its participation as a central modulator in the muscle-kidney crosstalk in CKD-associated muscle wasting.

## 2 Materials and methods

The experimental design of this work consists of two separated parts. 1) the role of exercise in mice submitted to a nephrotoxic experimental model and the effects of the blockage of STAT3 in these mice, and 2) The role of a newly identified molecule APLNR in CKD-associated muscle wasting in patients.

### 2.1 Animals

Male BALB/c mice from the animal care center of the Federal University of Parana were utilized in this study. Only male mice were used as the female mice are known to be resistant to DOX nephropathy injury [25,26]. Mice were housed in collective cage of up to 5 animals per cage at a 12-h light/dark cycle with a controlled temperature (22 ± 2 °C) and humidity and were fed with *ad libitum* access to water and food. Mice had 12 weeks of age at the start of exercise or sedentary protocol. Experimental protocols were approved by the Ethics Committee of the Federal University of Paraná/Biological Sciences Center (CEUA/BIO – UFPR) under accession number 1184 and are in accordance with the ARRIVE guideline for animal experimentation.

### 2.2 Drugs and experimental design

The experimental design of this study involved a resistance training protocol performed prior to any pharmacological intervention, followed by the induction of an experimental nephropathy model and modulation of STAT3 signaling in trained and untrained mice.

**Fig. 1:**
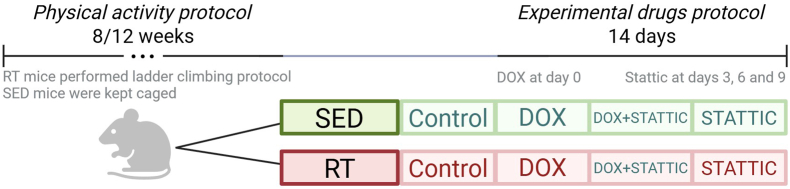
Experimental groups. This study consisted in 8 experimental groups. Mice were divided between two physical activity protocols: sedentary (SED) or resistance training (RT). After the exercise/sedentary protocol was finished, each protocol were subdivided into 4 sub-groups for the drug intervention protocols: Control, DOX, DOX + Stattic and Stattic.

Initially, mice were allocated into two main groups according to the physical activity protocol: (i) Sedentary (SED) or (ii) resistance training (RT). After completion of the resistance training protocol, animals from each group were randomly subdivided into four experimental subgroups based on nephropathy induction and STAT3 inhibition: (1) Control, (2) Stattic, (3) DOX, or (4) DOX + Stattic (Fig. 1). Each subgroup consisted of 6–7 mice.

Experimental nephropathy was induced by a single intravenous injection of doxorubicin hydrochloride (DOX) (Adriblastina®, Pfizer™, Inc. New York, USA) at a dose of 10 mg/kg via caudal vein [24]. To inhibit STAT3 signaling, the STAT3 activation and dimerization inhibitor STATTIC (Abcam ™, Inc., Cambridge, USA) was injected at 2 mg/kg in the caudal vein of mice in the days 3, 6 and 10 after DOX administration. Control mice had vehicle injections on the same days.

Fourteen days after DOX administration, animals were weighted, anesthetized (xylazine 16 mg/kg and ketamine 150 mg/kg) and euthanized by cardiac followed by cervical dislocation, with exception to the 12-week RT mice who were euthanized 28 days after DOX.

### 2.3 Resistance exercise protocol, adaptation and maximum weight carried test

Mice from RT protocol were submitted to an 8-week or 12-week training protocol of inclined ladder climbing with weights attached to the animals tails [27], while mice from SED protocol were kept caged for 8 weeks. Animals from the 8-week protocol stopped the RT training after completing 8 weeks of exercise and then received the drugs treatments, meanwhile mice from the 12-week protocol kept performing the RT protocol after receiving the drugs at week 8. The ladder parameters followed the description by Hornberger Jr & Farrar (2004) of which consisted in a 1.1 × 0.18 m ladder with steps separated by 2 cm and an inclination of 80° [27]. Animals from the RT protocol performed repeated whole-ladder climbing with fixed intervals between each climb.

Mice were first submitted to an adaptation period of 3 consecutive days, where they were allocated in a rest chamber at the top of exercise ladder for 2 min and then placed into the ladder from three different start-points: 35, 55 and 110 cm from the top, with no weight attached to its tail, and with 2 min interval between climbs [28,29]. After the adaptation protocol, mice were submitted to a maximum weight carried test (MWC) and started the 8/12-week RT protocol.

The MWC test, used to standardize the load between animals, consisted of a maximum of 6 climbs or until the animal could not reach 90% of total ladder height. Progressive heavier load was attached to mice tail in each try. For the first test, initial load was set at 75% of mouse body weight, with an increase of 5 g for each subsequent climb, and 120 s interval between climbs [30]. The last MWC value of each mouse was used as a base value for the subsequent tests. MWC test was performed at first, fourth and eight week of exercise protocol, and eleven days after Doxorubicin injection. Each new test started from 75% value of the last MWC as first load.

Mice then performed an 8/12-week exercise protocol, 3 days/week in intercalated days. Attached load ranges from 40% to 60% of the last MWC performed to represent a moderated intensity training load as recommended for chronic patients during exercise-treatment studies [31]. At each protocol session mice should perform a total of 20 climbing, with 90 s interval between attempts. After each two weeks of training the weight load was increased, from 40% to 50%, from 50% to a new MWC 50% and finally from 50% to 60% of the last MWC.

### 2.4 Kidney analysis

The left kidney was collected for hematoxylin and eosin (H&E) and picrosirius red staining for quantification of tissue lesion. After collection, kidney tissues were post-fixed in 4% paraformaldehyde at 4 °C for 24 h prior to routine histological processing. For H&E [32], twelve random fields of each slides were analyzed. Tubulo-interstitial fibrosis was analyzed by the expansion of the cellular matrix.

For the assessment of percentage renal fibrosis picrosirius staining was carried out. Tissue sections (5 μm) were mounted onto glass slides. Deparaffinization and rehydration were made with xylene (Merck) for 10 min (twice) and graded ethanol solutions (100%, 95%, 70%, Synth) for 5 min each, respectively. After resting, slides were immersed in picrosirius solution (0.1% Sirius red in picric acid, Merck) for 1 h at room temperature. Slides were rinsed briefly in distilled water and further in acidified water (0.5% acetic acid, Synth) for 2 min. Slides were dehydrated in graded ethanol solutions (70%, 95%, 100%, Synth) for 2 min each. Slides were cleared in xylene (Merck) for 5 min (twice) and mounted with coverslips using mounting solution (Sigma). The determination of fibrosis in situ was conducted through quantification of 10 to 13 fields at 200x total magnification using a fluorescence microscope (Leica DM1000) and acquisition on Leica Microscope Imaging Software (Leica corporation). The semi-quantification was performed on Image-J software. To minimize bias related to field selection and to account for differences in image availability across experimental groups, histological images were randomized at the analysis level. In some groups, multiple images were obtained from a single animal, whereas in others images were derived from more than one animal. Therefore, images from each experimental group were randomly assigned into two subsets using the Excel function RANDBETWEEN (1–2), ensuring that each group was represented by at least two independent mean values. This procedure enabled consistent statistical comparisons across groups while preserving within-group variability.

To delineate suspected fibrosis areas, the pathologist evaluated: (i) Tubular pathological features, including tubular fibrotic atrophy (characterized by flattened epithelium and thickened, wrinkled basement membranes); and Interstitial expansion, characterized by the widening of the spaces between tubules, often accompanied by matrix deposition; (ii) Glomerular alterations, such as focal or global glomerulosclerosis and periglomerular fibrosis (addressed by loss of structure and rupture of the glomerular capsule and glomerular hypercellularization). In addition, histopathological score was measured by analyzing 10 histological fields and counting the occurrence of histological alterations of: pyknotic nuclei, tubular cyst, brush border loss, tubular dilatation, glomerular matrix deposition, glomerular retraction, inflammation and loss of basal lamina.

### 2.5 Muscle analysis

Right gastrocnemius muscle was collected, measured and weighed, and let to rest for 15 min. After resting, the muscle was embedded in neutral talcum powder and frozen in liquid nitrogen. Semi-serial sections of 10 μm were cut at −20 °C using a cryostat. Histological staining techniques of hematoxylin and eosin [32], Sudan Black [33] histoenzymatic reaction of NADH-TR [34] were performed and photodocumented using microscope (Carl Zeiss™ Primo Star™) with a coupled camera a (Carl Zeiss™ AxioCam ERc 5s). ImageJ software [35] was used for histological analysis.

Fiber's classification of types I, IIa and IIb were performed through analysis of 4 histological fields per animal (100x magnification). Myofiber cross-sectional area (CSA) was measured using 50 fibers of each type per animal and 150 fibers of each type per group was selected through the *sample_n(.)* function available in the R statistical environment for the CSA comparison.

Morphometric and structural organization of muscle fiber were analyzed by counting of fibers, ratio of nuclei per fiber, presence of central nuclei and capillary per fiber through analysis of whole fibers present in 8 histological fields (400x magnification) per animal.

Intramuscular lipid deposition was analyzed using Sudan black staining technique, with 12 images (400x magnification) per animal through intensity of staining.

### 2.6 Albumin/creatinine ratio and serum parameters

Urinary albumin was estimated by SDS-PAGE electrophoresis at polyacrylamide gel concentration of 10%. Amersham Imager 600 from General Electric was used to quantify protein in gel bands. Creatinine from urinary samples was quantified using a colorimetric kit from Labtest Diagnóstica (Ref.: 35, Labtest, Lagoa Santa, MG, Brazil).

Serum samples were collected at the time of euthanasia and stored for analysis of urea, albumin and triacylglycerol. Serum urea was quantified using the commercial kit Uréia CE, Labtest Diagnóstica (Lagoa Santa, MG, Brazil), albumin was quantified using the commercial kit Albumin, Labtest Diagnóstica (Lagoa Santa, MG, Brasil) and the triacylgycerolemia was dosed by the commercial kit Triglicérides, Katal Biotecnológica (Belo Horizonte, MG, Brazil). All analysis were performed following the kit instruction.

### 2.7 Real time polymerase chain reaction

For quantitative real-time polymerase-chain reaction (qPCR), total RNA was extracted from frozen kidneys and gastrocnemius muscle (−80 °C) using TriZOL® Reagent (Thermo Fischer Scientific). cDNA was synthesized with previous DNase I, RNase-free (Thermo Fischer Scientific) treatment and posterior High-Capacity cDNA Reverse Transcription Kit (Thermo Fisher Scientific). The qPCR was performed with iTaq Universal SYBR® Green Supermix (Bio-Rad Laboratories).

The dCT value (Ct_Gene of interest_ – Ct_Reference Gene_) was used for statistical analysis and the relative expression (10000/2^dCT) for graphical expression. The mRNAs levels for kidney NPHS1, KIM1, and muscle FBXO32, MYOD1 and APLNR were analyzed; ACTB was used as reference gene. Primers sequences are described in Table S1 (Supplementary Information).

### 2.8 Data acquisition and expression analysis

Data from RNA sequencing of muscle from CKD patients was obtained through Gene Expression Omnibus (GEO, https://www.ncbi.nlm.nih.gov/geo/) platform. The GSE157712 (GPL20301) [36] was selected. To avoid interference of external variables, samples originating from dialytic patients were excluded from this analysis. The GSE99339 and GSE99325 (GPL19184) were used to validate the APLNR expression on kidney tissue. Data analysis was performed in R software (version 4.3) [37]. Data normalization was performed through “DESeq2” package. Enrichment analysis was performed with the “clusterProfiler” package and the GSEA collection was obtained through the MSigDB platform, accessed via “msigdbr” package. The packages “EnhancedVolcano” and “GOplot” were used for graphical expression of differential expression analysis and enrichment analysis, respectively.

### 2.9 Statistical analysis

Statistical analysis was performed using R software version 4.3.0 [37]. Data are presented as mean ± standard deviation or median (interquartile range). All data were initially tested for normality by the Shapiro-Wilk test. Mice data had the presence of outliers checked by the 1.5∗IQR rule and outliers were removed when parametric analysis were performed. For non-normal distributed data, comparisons between two groups used Mann-Whitney's *U* test. To test the distribution of muscle fibers, Kruskal-Wallis's with Dunn's post hoc test was performed. Friedman's repeated measure analysis was used for comparison of albuminuria over time. For normal distributed data and more than two groups the ANOVA test was performed. For repeated measures, only animals with complete data sets across all time points were included and a mixed ANOVA model with Greenhouse-Geisser correction for data sphericity and Bonferroni's *p* value correction was performed; and Pearson correlation was utilized in case of comparison among two variables. Statistical significance levels were set at α < 0.05.

## 3 Results

### 3.1 Blocking STAT3 signaling attenuated the magnitude of body weight loss in DOX-induce CKD mice after exercise

**Fig. 2:**
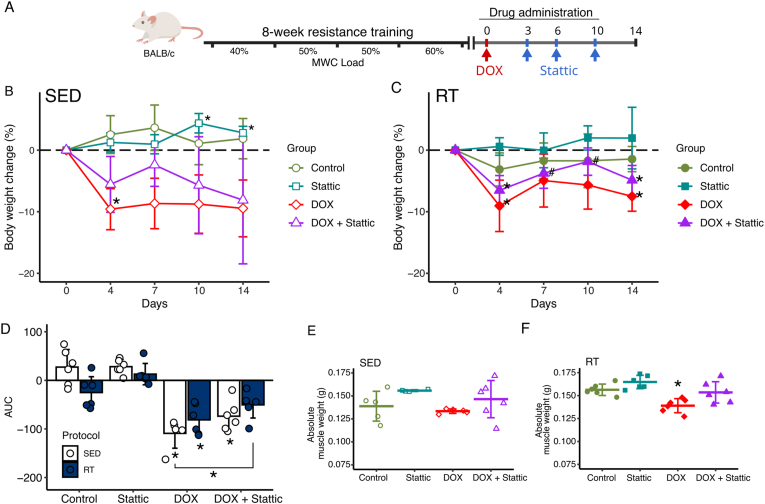
RT protocol and blocking STAT3 modulate body and muscle weight change over DOX administration. (A) Experimental design of exercise protocol indicating percentage of maximum weight carried (MWC) test load used at each two weeks of exercise protocol and drug administration during drugs protocol. The euthanasia occurred at 14 days after DOX administration. (B-C) Mean change in body weight of mice from SED and RT protocol over two weeks after drugs administration, Mixed ANOVA model for repeated measures (Group:Day p < 0.01 for both SED and RT protocols). ∗p < 0.05 vs Day 0, #p < 0.05 vs Day 4. (D) Area under curve of body weight variation, factorial ANOVA (Group:Protocol p-value < 0.01). ∗p < 0.05 vs control groups or between keys. (E-F) Absolute gastrocnemius weight of SED and RT protocols, factorial ANOVA (Group p-value <0.01. Protocol p-value < 0.01) ∗p < 0.05 vs control. Data expressed as mean ± standard deviation.

Body weight was measured at 0, 4, 7, 10 and 14 days after DOX administration, and the mass of the right gastrocnemius muscle measured on day 14 (Fig. 2). On day 4 after DOX administration, DOX mice in both SED and RT groups showed decreased body weight. Although mean body weight remained lower than baseline after Day 4, the high variability in individual recovery rates resulted in a loss of statistical significance relative to Day 0 at subsequent time points. The DOX + Stattic groups also experienced weight loss on day 4; however, the group from RT showed a temporary weight gain trend by day 10 compared to their peak loss, followed by a subsequent decline by day 14 (Fig. 2B–C).

Looking at the body weight data of area under curve we found that the DOX and DOX + Stattic mice from sedentary protocol had greater decrease of body weight through the drug protocol compared to Control and to DOX + Stattic from RT. Regarding RT protocol, only DOX mice had a significant body weight reduction when compared to the control group. Additionally, DOX + Stattic group from RT protocol had its weight loss partially mitigated when assessed by the area under curve, with lower body weight loss compared to the DOX-SED group and no difference to controls (Fig. 2 D). The Stattic and Control groups exhibited no changes in body weight, except for the expected increase due to normal animal growth.

Although DOX led to a decrease in absolute muscle weight (Fig. 2E–F), the relative muscle weight (adjusted for body weight) did not show significant changes (Table S2, Supplementary Information), possibly because overall body weight was also affected. Notably, despite the absence of muscle weight gains due to the exercise protocol, all trained mice showed improvement in individual performance, as assessed by the maximum weight carried test throughout the exercise intervention (Table S3, Supplementary Information). The improvement observed in the maximum weight carried test indicates enhanced muscle function. These results demonstrate that the RT protocol effectively increased muscle strength during the training phase. Coupled with our preceding findings on body weight maintenance through STAT3 inhibition along RT protocol, this intervention might improve muscle function through mechanisms other than increasing muscle mass, such as enhancing muscle strength and endurance. Finally, functional renal parameters were analyzed such as uremia, albuminemia and tracylglycerolemia and no differences between groups were detected (Fig. S1, Supplementary Information).

### 3.2 STAT3 inhibition safeguard and maintain the RT-driven kidney benefits after DOX-induced nephropathy

**Fig. 3:**
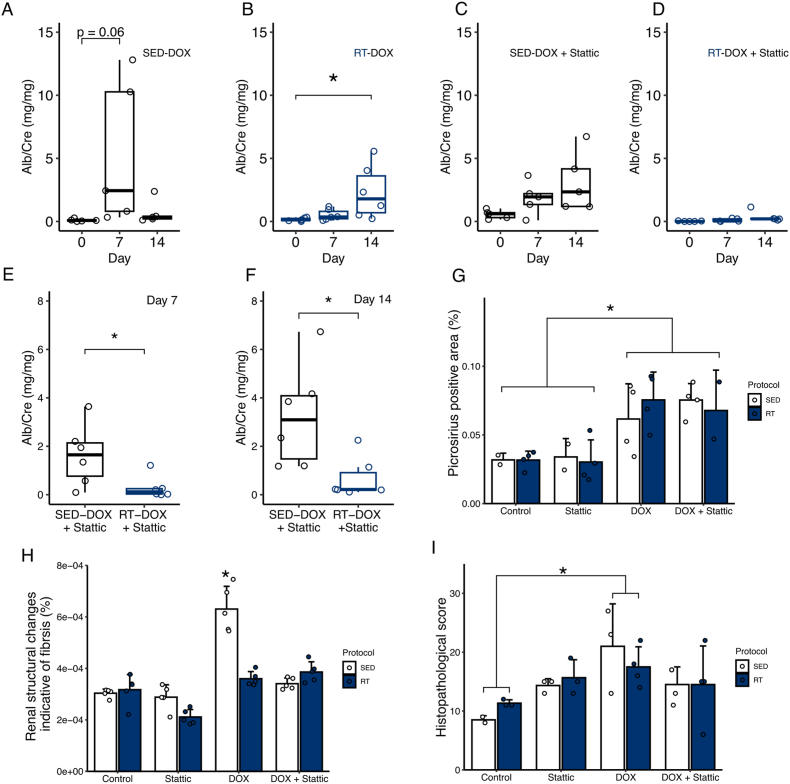
DOX-induced kidney injury induces albuminuria and histological lesion, while STAT3 inhibition reduces albumin to creatinine ratio. (A-D) Albumin/Creatinine (mg/mg) ratio through 14 days from DOX-injected mice from SED and RT protocols. Friedman’s repeated measure test. (E-F) DOX + Stattic albumin/creatinine levels after 7 and 14 days of DOX administration. Kruskall-Wallis test. (G-H) Histological analysis of the kidney tissue of picrosirius (factorial ANOVA. Group p-value < 0.001) and Hematoxylin and eosin staining of kidney tissue, factorial ANOVA (Group:Protocol p-value < 0.001). (I) Histopatological score of kidney tissue (factorial ANOVA, Group p-value < 0.05) Data expressed as median and interquartile range. ∗ p<0.05.

A significant increase in the urinary albumin to creatinine ratio, a classic biomarker of CKD severity, was detected in mice injected with DOX in both SED and RT protocols. Curiously, DOX mice from SED group had a detectable albuminuria at day 7, while this increase only occurred after 14 days for the RT group (Fig. 3A–B). Surprisingly, the RT-DOX + Stattic group did not exhibit any alteration in albuminuria, considering that a tendency of increasing was observed in SED-DOX + Stattic group (Fig. 3C–D).

Analysis of urine from Control and Stattic groups from both RT and SED protocols showed no increase in urinary albumin/creatinine ratio over time, although in day 7 a tendency of increase was observed in SED-DOX group (Fig. S2, Supplementary Information). Direct comparison of DOX groups of RT *vs* SED protocols revealed no difference in albuminuria (Fig. S2, Supplementary Information). Meanwhile, the DOX + Stattic mice achieved a reduction in urinary albumin/creatinine for RT group compared to SED (Fig. 3E–F). Representative SDS-PAGE gels are available in Supplementary Fig. S3.

Furthermore, by picrosirius histological analysis all DOX groups (with and without Stattic) had increased collagen deposition (Fig. 3 G), indicating that DOX administration leads to elevated matrix deposition in kidney tissue. Analysis of hematoxylin and eosin staining for renal structural changes indicative of fibrosis areas and histopathological scores were increased in DOX-treated groups, with no additional changes observed following Stattic administration (Fig. 3H–I). Representative histological images are available in Supplementary Fig. S4, and a table of parameters analyzed for histopathological score are discriminated in Supplementary Table S4 (Supplementary information).

### 3.3 Blocking STAT3 signaling and exercise protocol exhibits a synergism effect in the modulation muscle fibers population

**Fig. 4:**
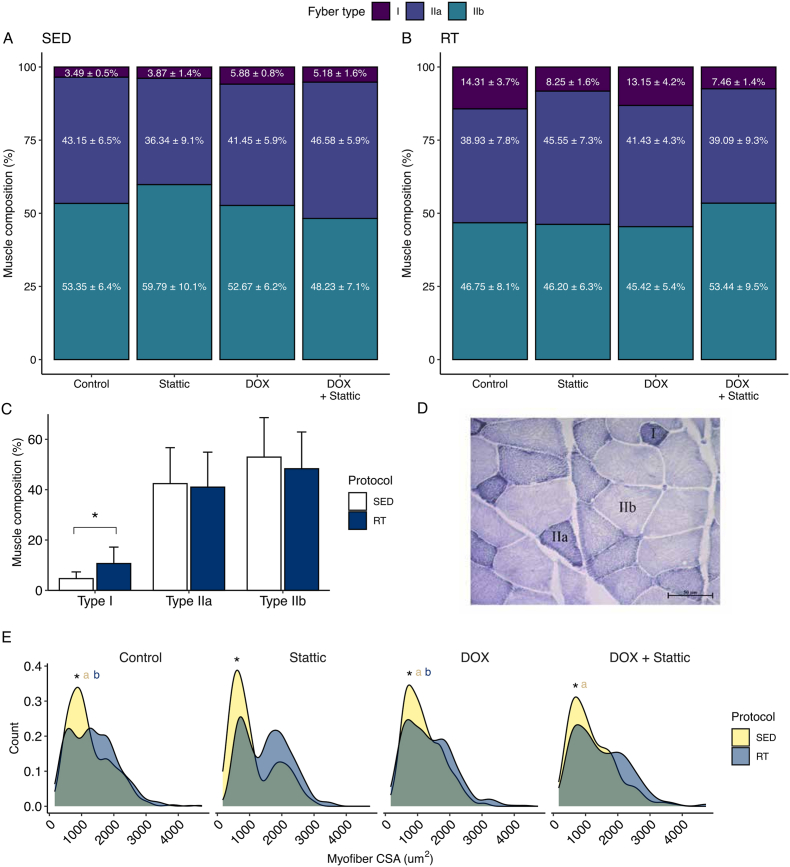
Muscle fiber distribution and classification after DOX administration along SED and RT protocols. (A-B) Gastrocnemius muscle composition stratified as fibers I, IIa and IIb of SED protocol and RT protocol. (C) Protocol effect on fiber distribution across all groups. Factorial ANOVA test (Protocol p-values < 0.001). (D) NADH-TR staining of gastrocnemius muscle. (E) Density plot of stratification of fibers by its cross-sectional area. Kruskal-Wallis test. ∗ p<0.05 SED vs RT; a p < 0.05 vs Stattic SED; b p < 0.05 vs Stattic RT. n = 3 to 5 mice per group. Data expressed as mean ± standard deviation.

Histological images of transverse cryosections of gastrocnemius with NADH-TR classified muscle fibers in types I, IIa and IIb (Fig. 4 D and Fig. S5, Supplementary Information) were assessed. The distribution of type IIa and IIb fibers was unbalanced in the presence of Stattic in sedentary animals, composed of 36.3% and 59.8% in sedentary mice, respectively (Fig. 4 A), whereas the resistance exercise protocol attenuated these variations, maintaining a distribution of more or less 45-46% of type II fibers (Fig. 4 B). Regarding fiber type alterations, we found that only the RT protocol promoted any effects, with resistance training increasing the proportion of type I fibers from 3.9% in sedentary group to 10.7% (Fig. 4C). Consistently, exercise promoted an increase in the proportion of larger muscle fibers, regardless of the pharmacological intervention. Notably, Stattic treatment was associated with a bimodal fiber size distribution, a pattern that was not observed when Stattic was combined with resistance exercise. While Control groups from SED and RT protocols reported a median fiber area of 1078 and 1293 μm^2^, respectively, the Stattic administration promoted changes in fiber distribution, with the sedentary Stattic group reporting a median value of 931 μm^2^, and 1543 μm^2^ for the RT protocol. DOX groups maintained values closer to the Control groups (Fig. 4 E).

### 3.4 STAT3 blockage prevents loss of exercise-induced morphometric muscle characteristics by DOX administration

**Fig. 5:**
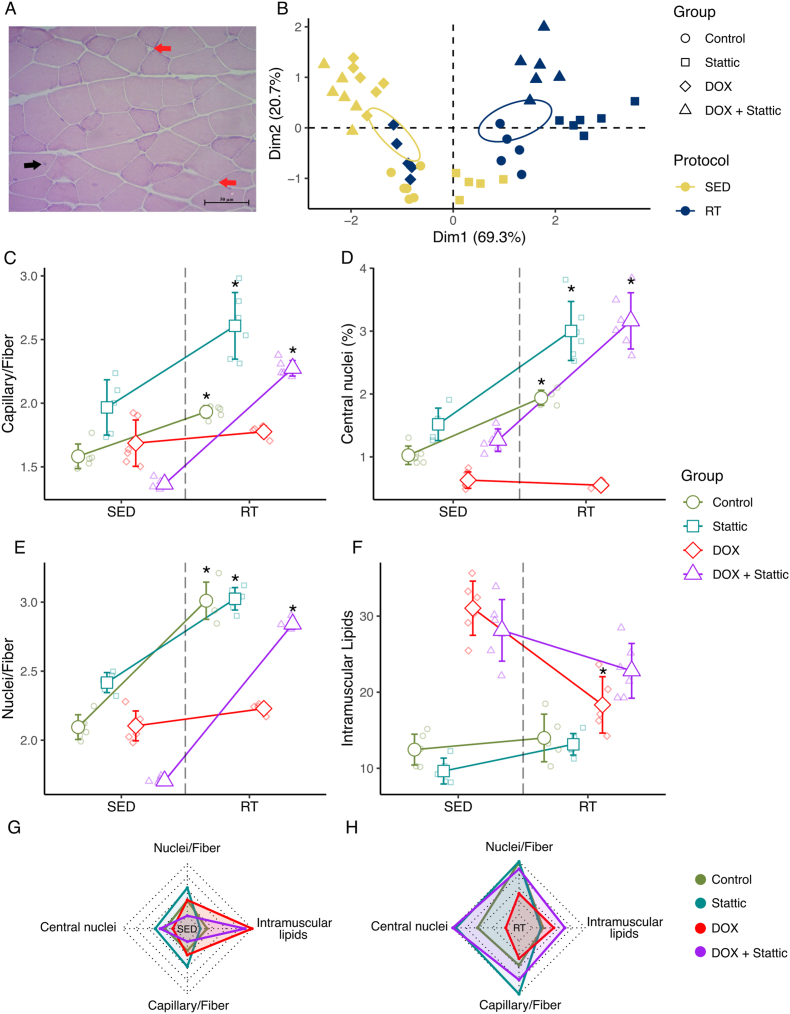
Morphometric characteristics of gastrocnemius muscle of mice from SED and RT protocols. (A) Histological image of gastrocnemius muscle in HE staining. Black arrow: Central nuclei. Red arrows: capillaries. (B) Principal component analysis of morphological characteristics. (C) Capillary/fiber ratio. (D) Percentage of centronucleated myofibers. (E) Nuclei/fiber ratio. (F) Intramuscular lipids score of sudan black staining. Factorial ANOVA (Group∗Protocol p-value < 0.001 for all variables. (G – H) Radar plot of mean values of morphological parameters of SED (left) and RT (right) protocols. ∗ p < 0.05 RT vs SED. Data were expressed by mean ± standard deviation.Gene expression profile of CKD patients over healthy individuals.

Morphometric characteristics from gastrocnemius muscle analyzed after hematoxylin and eosin staining (Fig. S6, Supplementary Information) showed that doxorubicin negatively affected muscle histological structure, while resistance training preserver or improved capillarity, myonuclear content, and lipid distribution (Fig. 5A–B; G-H). Morphometric indices typically enhanced by resistance training, including the capillary-to-fiber ratio, the proportion of centrally located nuclei, and the nuclei-to-fiber ratio in the gastrocnemius muscle, were increased in RT mice. However, these exercise-induced adaptations were blunted by doxorubicin administration, as no significant differences between RT and SED protocols were observed in the DOX group (Fig. 5C–F). In contrast, when STAT3 signaling was modulated, resistance training preserved these morphometric features despite DOX exposure, supporting a protective role of STAT3 blockade on exercise-induced muscle adaptations. Additionally, intramuscular lipid content was reduced in DOX-treated mice subjected to resistance training compared to their sedentary counterparts, whereas no significant protocol-related differences were detected in the remaining groups (Fig. 5C–H). Notably, the combination of resistance training and Stattic appeared to potentiate specific adaptations, suggesting a complementary rather than primary role for STAT3 inhibition under these experimental conditions.

### 3.5 Identification of differential expressed genes and functional enrichment pathways in the muscle of chronic kidney disease patients

Our findings demonstrated that the combination of resistance exercise training and blocking STAT3 signaling exerted beneficial impacts on morphological muscle characteristics, albuminuria and body weight in the experimental model of DOX-induced CKD. In attempt to gain deeper insights into the underlying molecular mechanisms in a human context, we carried out an extensive analysis with open access available gene expression data.

**Fig. 6:**
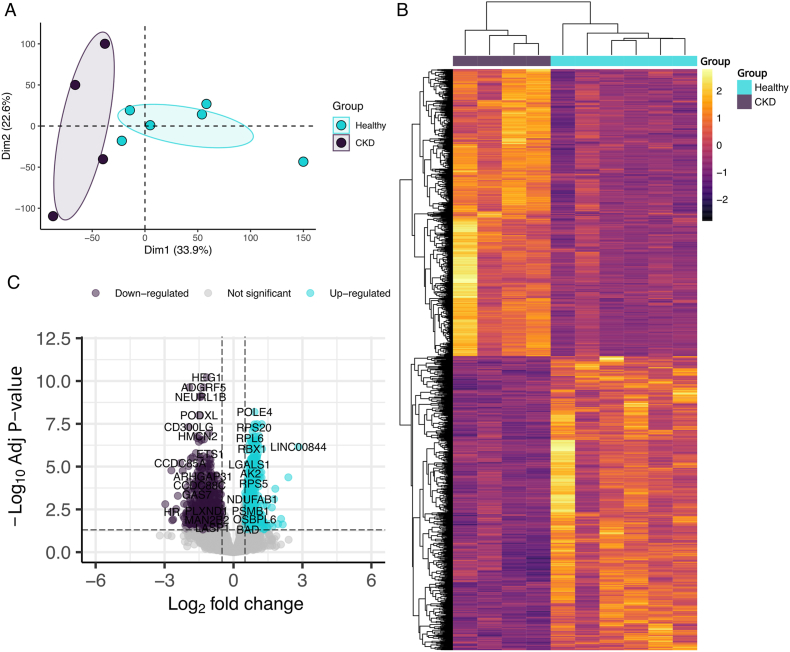
Gene expression profile of CKD patients over healthy individuals. (A) Principal component analysis and (B) heatmap of normalized expression of genes in the GSE157712 dataset. (C) Volcano plot showing differential expressed genes when |Log2 Fold Change| > 0.5 and adjusted p-values < 0.05.

We analyzed a GEO dataset (GSE157712) containing gene expression profiles of muscle tissue from both chronic kidney disease patients and healthy individuals [36]. Global profile of muscle samples reveal a distinct pattern of expression between healthy and CKD patients’ muscle tissue (Fig. 6A–B). A total of 717 down-regulated and 700 up-regulated genes were found as differentially expressed genes (DEGs) (Fig. 6C).

**Fig. 7:**
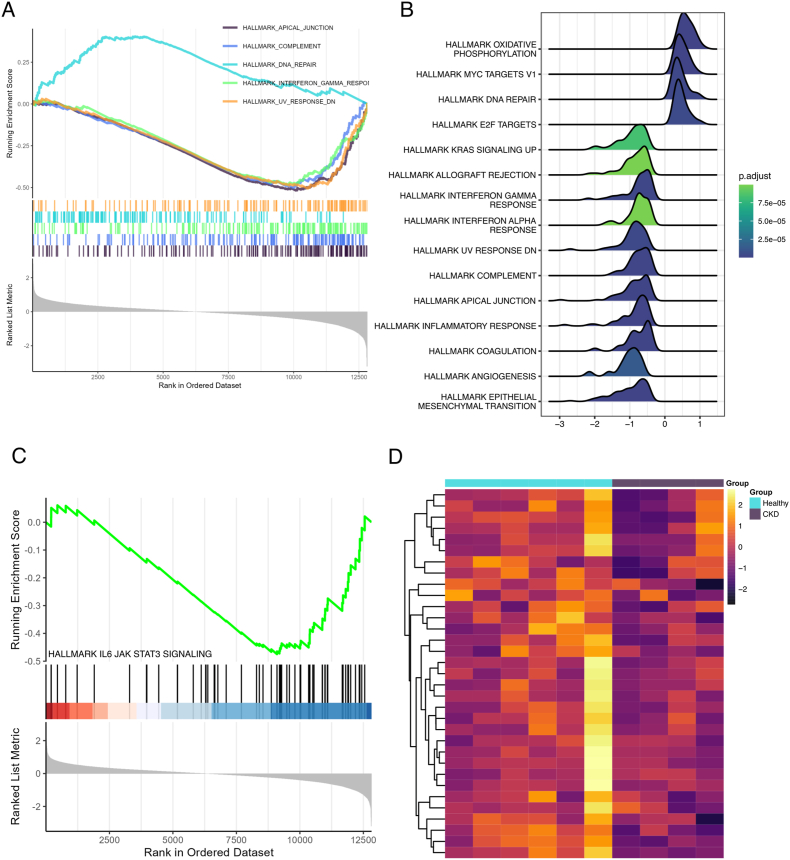
Gene set enrichment analysis in CKD patients and healthy individuals. (A) GSEA plot of 5 first hallmarks. (B) Ridgeplot of main hallmarks. (C) GSEA plot of IL6-JAK-STAT3 hallmark and (D) heatmap of normalized expression of genes of STAT3 hallmark present in the dataset. GSEA show altered inflammatory and angiogenic response in CKD patients.

In sequence, GSEA analysis was conducted on the whole set of genes ranked by its fold change value. Hallmarks of inflammation and angiogenic response were found to be enriched (Fig. 7A–B). The IL-6-JAK-STAT3 hallmark was also enriched among main terms investigated, and its genes expression profile was explored (Fig. 7C–D).

**Fig. 8:**
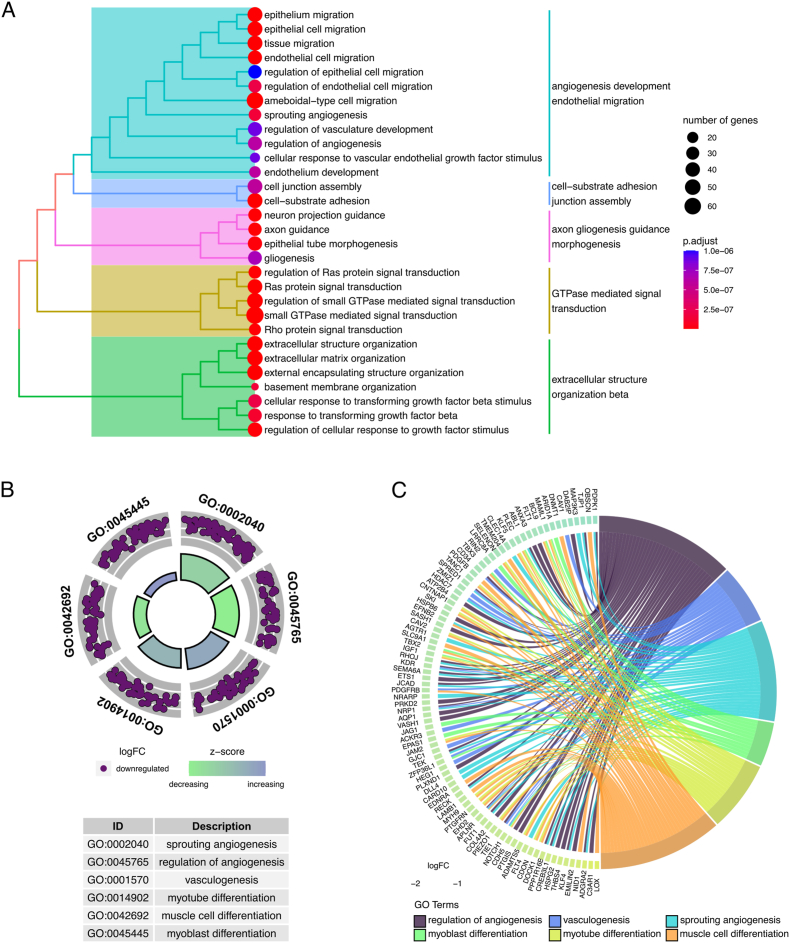
Gene ontology functional enrichment analysis of down-regulated genes among CKD and healthy individuals. (A) Treeplot of terms and its clustering, (B) Circleplot of selected GO terms and (C) Chordplot of genes present in selected GO terms and its connection with such terms. GO functional enrichment reveal common genes to angiogenesis and muscle differentiation.

In addition, GO functional enrichment analysis of down-regulated genes was performed and expressed as treeplot (Fig. 8 A) highlighting the contribution of genes to angiogenesis process. Moreover, analysis of selected enriched GO terms highlights the participation of key genes in processes related to muscle cell differentiation (*“myoblast differentiation”, “myotube differentiation”, “muscle cell differentiation”*) and vascularity (“*regulation of angiogenesis”, “vasculogenesis”, “sprouting angiogenesis”*) (Fig. 8B–C).

### 3.6 APLNR acts as a key gene associated with modulation of vascularity and muscle cell differentiation in muscle of chronic kidney disease patients and mice from DOX-induced kidney injury

**Fig. 9:**
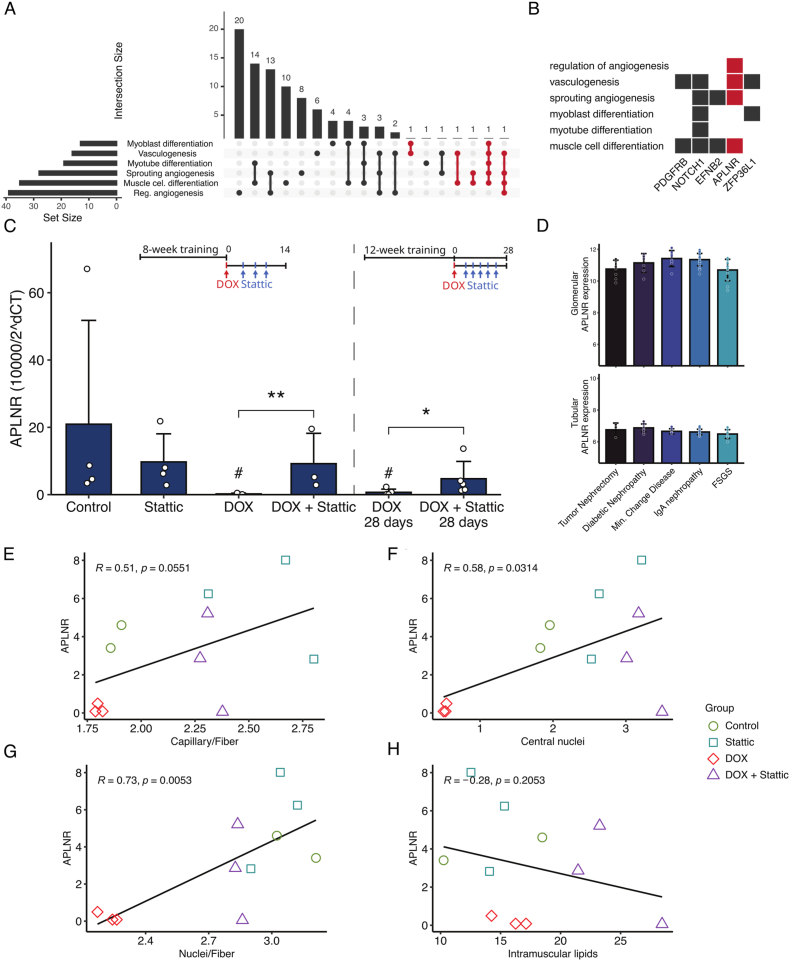
APLNR gene as a key component of enriched terms and its expression in exercised muscle. (A) Upset plot illustrates the intersection of genes between selected significant GO terms of musculature differentiation and vascularity processes. Red lines indicate unique genes to more than one term of each class. (B) Unique genes selected and their participation on the different GO terms. (C) APLNR mRNA expression on muscle of mice of two distinct protocols of exercise and STAT3 inhibition (see protocols above the bars). ANOVA test (D) Glomerular and tubular expression of APLNR in different kidney pathologies. (E-H) Pearson correlation of histological alterations and muscle expression level of APLNR of RT mice. FSGS: Focal Segmental Glomerulosclerosis. ∗p<0.05, ∗∗p<0.001, # p<0.05 vs Control and Stattic.

To further explore the potential key regulators of vascular and muscle differentiation processes in the muscle during CKD, we aimed at the genes simultaneously present in at least one main GO term related to muscle differentiation and one principal GO term related to angiogenesis/vascularization (Fig. 9 A). The candidate genes were PDGFRB, NOTCH1, EFNB2, APLNR and ZFP36L1. The APLNR gene was overrepresented in most terms, and it was selected for analysis in our *in vivo* model (Fig. 9 B). We measured APLNR in the muscle of exercised mice from two distinct protocols differing in the RT-protocol duration and exposure to DOX: 1) 8 weeks of RT protocol with exercise interruption before drug administration, and 2) 12 weeks of RT protocol without interruption of exercise during the drugs administration period. In both cases, DOX groups had lower muscle expression of APLNR, while treatment with Stattic prevented this effect (Fig. 9C). Expression of APLNR in sedentary mice did not change (Fig. S7, Supplementary Information), as so MYOD1 and FBXO32 (Fig. S8, Supplementary Information).

Gene expression of APLNR in human kidney was also evaluated using another dataset (GSE19184). No differences in kidney APLNR expression was found in individuals from clinical diagnostic of diabetic nephropathy, minimal change renal disease, IgA nephropathy or FSGS when compared to kidney tumor nephrectomy (control sample) (Fig. 9 D). These data indicate that APLNR expression is not markedly altered in the kidney during the course of kidney disease; however, its modulation in skeletal muscle may increase sarcolemmal APLNR availability, thereby enhancing responsiveness to myofiber-derived apelin and potentiating local autocrine/paracrine APLN–APLNR signaling.

Then, trained mice had its APLNR expression correlated to its muscular morphohistological alterations of capillarity, central nuclei, nuclei-to-fiber ratio, and intramuscular lipids scores (Fig. 9E–H). We found mild to strong positive correlations of APLNR expression in the muscle and morphological alterations of capillarity and nuclei parameters, except for intramuscular lipids that showed weak negative correlation.

Furthermore, a PPI network was constructed using the STRING database to verify the interaction between the proteins STAT3 and APLNR (Fig. S9, Supplementary Information). We show that the STAT3 protein is linked indirectly to APLNR through OPRD1, an important signaling G-protein coupled receptor.

## 4 Discussion

In this work, we provided data on how RT and STAT3 blockage improved muscle response under the nephrotoxic model of doxorubicin and found, through computational analysis of a RNA-seq dataset, the APLNR gene to be a key regulator of muscle response during CKD. We later measured the levels of APLNR in the muscle of our experimental model to find that in mice with kidney injury the levels of APLNR are reduced, but could be rescued by RT when the STAT3 is inhibited during the onset of the lesion. Our findings suggest that the observed muscular downregulation of the APLNR axis is a downstream consequence of this maladaptive kidney-muscle crosstalk.

The protective effects of physical exercise to chronic patients, including CKD, have been explored over the years along with the importance of the STAT3 signaling pathway on muscle catabolism [10] and damage to the kidney tissue [38]. STAT3 activates in response to muscle activity in a physiological response to stimuli, however, the continuous IL-6 activation of STAT3 in pathological condition is linked to muscle atrophy responses, as those observed in cancer cachexia [11].

We observed a DOX-induced significant weight loss independently of exercise protocol, which was partially mitigated in trained mice with blocking of STAT3 signaling. DOX administration leads to a body mass loss over time and can be used as a clinical marker for development of renal injury in BALB/c mice [24]. Weight loss in DOX induced nephropathy is present at even longer time-spam, with no total recovering (body weight matching pre-drug period) over the course of up to five weeks [39]. Analysis of the gastrocnemius muscle weight revealed a loss of muscle mass due to DOX when comparing groups from the RT protocol. Similar to our findings, others identified that mice soleus weight did not decrease after a model of DOX chemotherapy and endurance training protocol [40]. In contrast, a systematic review of muscle effects of DOX experimental models reported a skeletal muscle weight reduction of 14% (95% CI: 9.9 to 19.3) to different muscles, including to mice gastrocnemius [41]. These reports suggest that muscle weight measurement may have discrepancies depending on the protocol adopted and it does not appear to be a reliable parameter if analyzed singly, requiring interpretation in conjunction with other attributes related to the muscle-associated disease.

Resistance training is known to exert beneficial effects on skeletal muscle quality. Resistance training induces well-characterized neuromuscular adaptations that enhance force production and muscle quality even in the absence of hypertrophy. Chronic exposure to resistive loading increases neural drive, motor unit recruitment, and muscle coordination, contributing to improved strength outcomes and functional capacity [42]. Exercise also exerts systemic metabolic effects by modulating muscle and whole-body metabolism, including improvements in glucose uptake and lipid handling, which have been observed in both rodent models and clinical populations with CKD [43].

Although no increase in absolute or relative muscle mass was observed, the improvement in muscle performance suggests functional adaptations independent of hypertrophy. Increases in muscle strength without changes in muscle size are well described and are largely attributed to neural adaptations, including enhanced motor unit recruitment, increased discharge rates, and improved neuromuscular efficiency. Additionally, improvements in excitation–contraction coupling and force production per unit of muscle cross-sectional area (specific force) may contribute to greater functional output without detectable hypertrophy. This dissociation between strength and size has been documented in both human and animal studies, and highlights how neural and motor control mechanisms contribute to strength gains in the absence of hypertrophy. Such mechanisms are particularly relevant in pathological conditions or short-term interventions where morphological changes are limited but functional improvements occur [[44], [45], [46]].

Additionally, in our study, DOX raised urinary albumin to creatinine index levels that were lower after exercise or STAT3 inhibition independently. Moreover, we noticed that the combination of RT with STAT3 inhibition greatly reduced the albuminuria levels promoted by DOX. DOX administration in BALB/c mice may cause a two-phased lesion, where the glomeruli first suffer from loss of its macromolecular filtration capacity, leading to proteinuria, to later become sclerotic with reduced filtration surface availability, causing the lowering of proteinuria levels [39]. An earlier detectable peak of albuminuria at day 7 with no albumin/creatinine at day 14 may be due to an aggressiveness of the model at mice with no co-therapy (SED-DOX), since SED-DOX + Stattic did not result in detectable albuminuria (p > 0.05) compared to the fact that RT-DOX mice did perform a progressive increase in proteinuria that was detected at day 14. Surprisingly, this negative effect of DOX on renal function was abolished when the inhibition of the STAT3 pathway was carried out concomitantly with RT. An experimental model of lupus nephritis in mice showed a delay of four weeks to the detection of albuminuria due to STAT3 blocking [47], indicating that the blocking of STAT3 signaling may promote renoprotection.

Souza et al. (2018) found that RT exercise leads to reversion of renal fibrosis in comparison to SED nephrectomized rats in a 5/6 nephrectomy model. Furthermore, the authors demonstrated that STAT3 inhibition not only reduces urinary protein levels but also protects against renal fibrosis, tubular necrosis and inflammatory infiltration [48]. In our study, picrosirius positive area staining on kidneys revealed increased collagen deposition on mice from DOX groups, while we detected augmented fibrotic area in kidneys from sedentary DOX mice only through HE staining. Further analysis with different histological staining techniques or by molecular analysis of fibrosis components could elucidate the magnitude of such tissue alterations.

DOX-induced skeletal muscle myopathy can be observed in some mice models. Along with weight reduction, DOX causes loss of twitch force and reduction in muscle fiber cross-sectional area [41]. Testa et al. (2022) reported that exercise may partially reverse this effect where an impaired locomotor capacity and muscle atrophy were prevented by RT.

Complementarily, RT prevented the increase in expression of molecules associated with STAT3 phosphorylation and muscle atrophy [11]. Our results showed that resistance training promotes histological alterations in the gastrocnemius muscle, improving capillarity and myonuclear characteristics, including centralized nuclei and the nucleus-to-fiber ratio. All histomorphometric characteristics derived from previous exercise protocol were severely affected after DOX administration, which was reverted by the combination of RT protocol and STAT3 inhibition.

Rodent skeletal muscle fibers have different myosin heavy chain types and oxidative capacity, being classified for its myosin heavy chain isoform as types I (oxidative, slow-twitch), IIa (oxidative, fast-twitch) and IIb (glycolytic, fast-twitch), with fiber type switching occurring in response to exercise, nerve stimulation and hormonal influence [49,50]. Our findings showed that RT protocol causes alterations on fiber distribution according to its cross-sectional area, where the Stattic administration promoted major contributions. Tierney et al. (2014) found, in the context of acute injury (5 days after lesion), that the inhibition of STAT3 increased higher sized myofibers population, responsible for an accelerated tissue repair process [51]. Furthermore, Gehlert et al. [52] found that resistance exercise was able to promote an increase in the diameter of type I and type II fibers after five weeks of resistance training.

Moreover, we also identified that the capillary/fiber ratio maintained the improvement due to RT in mice that received DOX due to the combination of RT and Stattic administration. In response to physical exercise stimuli the skeletal muscle capillarity increases [50]. DOX administration results in a reduction of capillarity of skeletal muscle [53] with satellite cells as mediators in the regenerating muscle contributing with capillaries and capillary growth. Activated satellite cells are known to secrete angiogenic factors, particularly VEGF, thereby stimulating endothelial cell proliferation and supporting microvascular homeostasis during tissue repair [54]. In this manner, the observed increase in capillarity of RT-Stattic group may reflect the functional mechanisms of satellite cell repairing, since transient STAT3 inhibition leads to an augmented proliferation capacity of satellite cells [51]. Indeed, the satellite cell population is decreased in CKD patients compared to healthy individuals, and its associated role of sustaining microvasculature, the total capillarity and the distance of satellite cells to the nearest capillary, are found reduced during CKD, with a correlation between poor muscle capillarity and reduced eGFR [36].

In our study, we detected the STAT3 inhibition effects on capillarity to prevail against the depletory DOX effect, since higher capillary/fiber was found in RT DOX + Stattic compared to SED mice, while only mice from DOX group did not maintain this structural change. These findings suggest that transient STAT3 inhibition may mitigate DOX-induced alterations in muscle capillarity, which are linked to worsened renal function and skeletal muscle integrity. Mechanistically, STAT3 signaling was shown to regulate the expression of multiple pro-angiogenic mediators, including VEGFA and VEGFB, VEGFR2, MMP-2, MMP-9, IGF-1, and b-FGF, in addition to modulating cyclin D1 levels and cell-cycle progression in both endothelial and stromal compartments [55]. Although STAT3 is a canonical activator of VEGF under physiological conditions, its chronic hyperactivation in CKD promotes a fibrotic and inflammatory microenvironment that hinders effective regeneration [10,11]. Thus, our data indicate that inhibiting this aberrant STAT3 signaling prevents catabolic remodeling, indirectly restoring a muscle niche permissive to physiological angiogenesis.

Similarly, in response to RT, the nuclei/fiber ratio increases due to an augmented proliferation and fusion of satellite cells [56], and centralnucleated myofibers population increase in regenerative muscle sites [57]. The increased central nuclei observed to RT-DOX + Stattic group reflect the capacity of STAT3 blockage to promote greater tissue repair, as also observed in the context of acute injury by Tierney et al. [51]. Also, the intramuscular lipids accumulation was higher in the DOX groups, although only the RT group had a decrease of intramuscular lipids. With a small number of sample, Keddar et al. (2020) reported an association of kidney failure and fat accumulation in the skeletal muscle that was reversed after kidney transplantation, indicating that kidney disfunction contributes to ectopic fat redistribution [58].

Taken together, these findings highlight the role of STAT3 in both exercise preconditioning effects and DOX model of nephropathy, and that resistance exercise combined with inhibition of STAT3 signaling promotes protection against DOX induced nephropathy and the myopathy observed in this model.

To better understand the muscle-kidney interrelationship, we conducted a computational analysis on a muscle dataset from CKD patients. We found two distinct global expression patterns differing between CKD and Healthy individuals. The GSEA was performed and revealed hallmarks of inflammatory response, angiogenesis and STAT3 signaling to be regulated during CKD. Further analysis performed by GO highlighted the role of down-regulated genes in the processes of angiogenesis and muscle differentiation.

The absence of an appropriate supply of oxygen to the skeletal muscle leads to muscle wasting [59], and the reduced numbers of capillaries in the muscle of CKD patients [36] may affect cellular components of the skeletal muscle to induce mechanism of hypoxia-related muscle impairment [60]. As observed to the muscle of CKD patients from the investigated dataset, the terms related to regulation of angiogenesis/vascularization along with the terms related to muscle cells differentiation could reflect mechanisms of muscle loss associated with poorer capillarity in this tissue.

To further comprehend such mechanisms, we selected genes that were present in at least one GO term related to angiogenesis and one term related to muscle differentiation to proceed with further investigation. The APLNR, apelin receptor, was selected for *in vivo* validation analysis due its possible role in processes of kidney and muscle damage during CKD [22,23,61,62].

The role apelin system in muscle damage during CKD has been recently explored. Enoki et al. (2022) found both apelin and its receptor (APLNR) to be reduced in the skeletal muscle in CKD, although apelin serum concentration did not had lower levels, suggesting a local autocrine function in the muscle. Controversially, knockdown of apelin in the skeletal muscle of mice resulted in reduction of exercise-induced apelin in the plasma, although basal values of a resting state did not change [20]. Our results showed that the APLNR expression in the muscle suffer from DOX and had its exercise-induced expression inhibited, which was recovered when STAT3 was blocked in RT, while no differences of APLNR expression in either glomeruli or tubule tissue were found.

Activation of the apelin system by exercise is credited to reduce sarcopenia, enhance myogenic differentiation and to promote activation of muscle stem cells for regeneration processes [20]. While APLNR expression is well-documented in adult myofibers and endothelial cells [20], its presence in the specific niche of muscle Pax7+ stem cells remains controversial. Some authors report an increased level of APLNR, while others report no detection of the receptor in these cells [63], although resistance training promotes increase in apelin plasma concentration and apelin and APLNR mRNA levels in the skeletal muscle of middle-aged healthy individuals [64].

APLNR is present in endothelial cells of the injured muscle tissue in mice, but is not detected in muscle stem cells. Furthermore, the apelin mRNA was found to colocalizate within the blood vessels in TA muscles of mice, reinforcing the paracrine role of the APLNR axis to the muscle tissue [63]. Individuals with CKD muscle impairment or sarcopenia show endothelial dysfunction [65], with reduced capillarity in the affected muscle [36]. The treatment of distinct muscle dysfunction mice models with Apelin-13, the smallest active form of apelin, could reduce the progress of muscle dysfunctioning while promoting the number of CD31^+^ cells [63].

In fact, it is reported that STAT3 activation by IL-6 and consequent binding to an apelin promoter region leads to increased apelin levels and activity [14]. The relationship between STAT3 signaling pathway and the APLNR is still unclear and need further studies for complete elucidation. Other evidences show that APLNR overexpression reduces the activation of STAT3 in cardiomyocites after stroke [16] and elabela administration (another APLNR ligand) affects the phosphorylation of STAT3 in the cardiac tissue of hypertensive mice [15], while APLNR overexpression in ovarian cancer cells leads to increased STAT3 phosphorylation [18]. To gain insight on the relationship of STAT3 and APLNR, we conducted an empirical analysis based on de STRING database to perform a protein-protein interaction network. We found STAT3 to be closely, but indirectly, linked to APLNR through OPRD1, an important signaling G-coupled protein receptor. Although a single study has identified a clear association between OPRD1 gene and Kidney pathology (renal clear cell carcinoma), an exact crosstalk of this receptor with potential CKD-associated signaling pathways need to be further investigated [66].

In this context, we document a relation of STAT3 pathway and APLNR expression in the muscle, where STAT3 inhibition causes APLNR overexpression during DOX-mediated model of kidney lesion. This suggest that the APLNR expression in the muscle may be related to STAT3 signaling resulting in muscle physiological dynamic changes, which were improved by synergism with exercise. In light of the mechanisms of APLNR axis in the recent years, we now provide evidences that regulation of APLNR could be achieved by modulation of the STAT3 pathway and that the muscle impairment present in individuals with CKD may be susceptible to interventions aiming the APLNR/Apelin axis. However, further analysis is required to elucidate the precise mechanisms behind STAT3 regulation and the apelin system in context of muscle stimulation. Different animal models of kidney disease, as well as a wider sample size, may help to elucidate these relationships. We address that the evaluation of expression of APLNR ligands, Apelin and Elabela, as well as the direct measurement of the proteins involved in the STAT3 and APLNR pathways by appropriated techniques were not performed and are limitations of this study and we encourage future studies to highlight such details.

In conclusion, our findings demonstrated the muscle tissue function is affected during CKD, which can be attenuated by a protective and synergistic effect of resistance exercise and STAT3 inhibition. Furthermore, APLNR appears as a key gene associated with muscle dysfunction in CKD patients and its muscle expression can be regulated by resistance exercise and STAT3.

## Authors contributions

Gabriel Pereira: Conceptualization, Methodology, Formal analysis, Investigation, Writing – Original Draft, Visualization. Thabata Caroline de Oliveira Santos: Investigation. Sofía Tomaselli Arioni: Investigation. Pietra Mancini Seibt: Investigation. Luana Fortuna: Investigation. Emily Pereira dos Santos: Investigation. Rodrigo Lazzarotto: Investigation. Matheus Felipe Zazula: Investigation. Juan Sebastian Henao Agudelo: Resources, Writing – Review & Editing. Débora Tavares de Resende e Silva: Resources, Writing – Review & Editing. Elizabeth Cristina Perez Hurtado: Resources. Katya Naliwaiko: Resources, Writing – Review & Editing. Ricardo Fernandez: Validation, Resources, Writing – Review & Editing. Danilo Cândido de Almeida: Validation, Resources, Writing – Review & Editing, Supervision. Rafael Luiz Pereira: Validation, Resources, Writing – Review & Editing, Supervision, Funding acquisition.

## Ethics approval

This study was performed in line with the ARRIVEL guideline for *in vivo* experiments and was approved by the Ethics Committee of the Federal University of Paraná/Biological Sciences Center (CEUA/BIO – UFPR) under accession number 1184.

## Funding

This work was supported by Coordenação de Aperfeiçoamento de Pessoal em Nível Superior (CAPES), Conselho Nacional de Desenvolvimento Científico e Tecnológico (CNPq) and Fundação da Universidade Federal do Paraná (FUNPAR). We also would like to thank all technician and student that direct and indirectly contributed to experiments and images acquisition (Aparecida Maria da Gloria, Clara Versolato Razvickas and Adrieli Silva).

## Declaration of competing interest

The authors declare that they have no known competing financial interests or personal relationships that could have appeared to influence the work reported in this paper.

## Data Availability

Data will be made available on request.
