# Supplementary material for: APLNR reduction in kidney-muscle crosstalk in renal model recovered by exercise and STAT3 inhibition
Source: Biochem Biophys Rep. 2026 Apr 13;46:102565. doi: 10.1016/j.bbrep.2026.102565 (PMC13092669; doi:10.1016/j.bbrep.2026.102565)
Supplement: Multimedia component 1 [file mmc1.docx]

**Table S1. Sequences of qPCR Primers.**

| Primer | Forward 5`- 3` | Reverse 3`- 5` |
| --- | --- | --- |
| NPHS1 | ACCTGTATGACGAGGTGGAGAG | TCGTGAAGAGTCTCACACCAG |
| KIM1 | ACATATCGTGGAATCACAACGAC | ACAAGCAGAAGATGGGCATTG |
| FBXO32 | GTGGCATCGCCCAAAAGA | TCTGGAGAAGTTCCCGTATAAGT |
| MYOD1 | ACGACTGCTTTCTTCACCACTCCT | TCGTCTTAACTTTCTGCCACTCCG |
| APLNR | CCAGTCTGAATGCGACTACG | CTCCCGGTAGGTATAAGTGGC |
| ACTB | *CTGTATTCCCCTCCATCGTG* | *GGGTCAGGATACCTCTCTTGC* |

Table sequence of primers used in the quantitative PCR assays.

**Table S2. Relative muscle weight of right gastrocnemius.**

| **Group** | **Sedentary (mg/g)** | **Resistance Training (mg/g)** | **p-value** |
| --- | --- | --- | --- |
| Control | 4.00 | 5.31 | > 0.05 |
| Stattic | 5.52 | 4.78 | > 0.05 |
| DOX | 5.18 | 4.88 | > 0.05 |
| DOX + Stattic | 5.13 | 5.12 | > 0.05 |

Relative muscle weight of right gastrocnemius muscle to body weight of mice from both protocols and all experimental groups.

**Table S3. Mean of Maximum weight carried by mice of all groups submitted to the resistance exercise protocol.**

| Test^#^ | Maximum weight (g) |
| --- | --- |
| 1^st^ | 37,6 ± 1,04ª |
| 2^nd^ | 40,8 ± 1,11^b^ |
| 3^rd^ | 47,4 ± 2,02^c^ |
| 4^th^ | 48,3 ± 1,26^c^ |

Results of maximum weight carried test of mice from resistance training protocol over the weeks. #*Tests were performed at weeks 1, 4, 8 and eleven days after end of exercise protocol. Different letters represent significant statistical differences between groups (p<0.05).*

| **Table S4. Parameters of histopathological score for analysis of kidney tissue.** | | | | | | | | | | |
| --- | --- | --- | --- | --- | --- | --- | --- | --- | --- | --- |
| **Group** | **Protocol** | **Picnotic Nuclei** | **Tubular Cyst** | **Brush Border Loss** | **Tubular Dilatation** | **Glomerular Matrix deposition** | **Glomerular Retraction** | **Inflammation** | **Loss of Basal lamina** | **Total**  **score** |
| DOX | RT | 3 | 2 | 1 | 3 | 0 | 2 | 0 | 5 | 16 |
| DOX | RT | 4 | 6 | 3 | 3 | 0 | 0 | 0 | 6 | 22 |
| DOX | RT | 0 | 5 | 1 | 3 | 0 | 0 | 0 | 5 | 14 |
| DOX | RT | 3 | 2 | 3 | 2 | 0 | 0 | 3 | 5 | 18 |
| DOX | RT | 0 | 0 | 0 | 0 | 0 | 0 | 0 | 0 | 0 |
| DOX + Stattic | RT | 5 | 0 | 1 | 3 | 0 | 1 | 0 | 5 | 15 |
| DOX + Stattic | RT | 2 | 0 | 1 | 5 | 1 | 0 | 0 | 6 | 15 |
| DOX + Stattic | RT | 5 | 3 | 5 | 3 | 0 | 0 | 1 | 5 | 22 |
| DOX + Stattic | RT | 2 | 0 | 2 | 2 | 0 | 0 | 0 | 0 | 6 |
| Stattic | RT | 3 | 0 | 1 | 3 | 0 | 2 | 2 | 2 | 13 |
| Stattic | RT | 5 | 0 | 1 | 1 | 0 | 0 | 0 | 8 | 15 |
| Stattic | RT | 2 | 0 | 1 | 7 | 0 | 0 | 2 | 7 | 19 |
| Control | RT | 3 | 1 | 1 | 3 | 0 | 0 | 1 | 2 | 11 |
| Control | RT | 3 | 1 | 1 | 3 | 0 | 0 | 2 | 2 | 12 |
| Control | RT | 3 | 1 | 1 | 1 | 0 | 1 | 1 | 3 | 11 |
| DOX | SED | 7 | 0 | 1 | 7 | 0 | 2 | 3 | 7 | 27 |
| DOX | SED | 3 | 2 | 1 | 0 | 0 | 0 | 2 | 5 | 13 |
| DOX | SED | 8 | 2 | 1 | 5 | 0 | 0 | 2 | 5 | 23 |
| DOX + Stattic | SED | 1 | 0 | 1 | 1 | 0 | 0 | 0 | 8 | 11 |
| DOX + Stattic | SED | 7 | 3 | 1 | 1 | 0 | 0 | 0 | 5 | 17 |
| DOX + Stattic | SED | 7 | 0 | 1 | 0 | 0 | 0 | 0 | 5 | 13 |
| DOX + Stattic | SED | 3 | 0 | 1 | 8 | 0 | 0 | 0 | 5 | 17 |
| Stattic | SED | 0 | 2 | 1 | 2 | 0 | 0 | 0 | 8 | 13 |
| Stattic | SED | 0 | 0 | 0 | 0 | 0 | 0 | 0 | 0 | 0 |
| Stattic | SED | 0 | 2 | 1 | 7 | 0 | 0 | 0 | 5 | 15 |
| Stattic | SED | 0 | 0 | 1 | 5 | 0 | 2 | 2 | 5 | 15 |
| Control | SED | 2 | 0 | 1 | 2 | 0 | 0 | 0 | 3 | 8 |
| Control | SED | 0 | 0 | 0 | 0 | 0 | 0 | 0 | 0 | 0 |
| Control | SED | 0 | 0 | 1 | 5 | 0 | 0 | 0 | 3 | 9 |
|  |  |  |  |  |  |  |  |  |  |  |

Histopathological scores of kidney slides from all experimental groups.


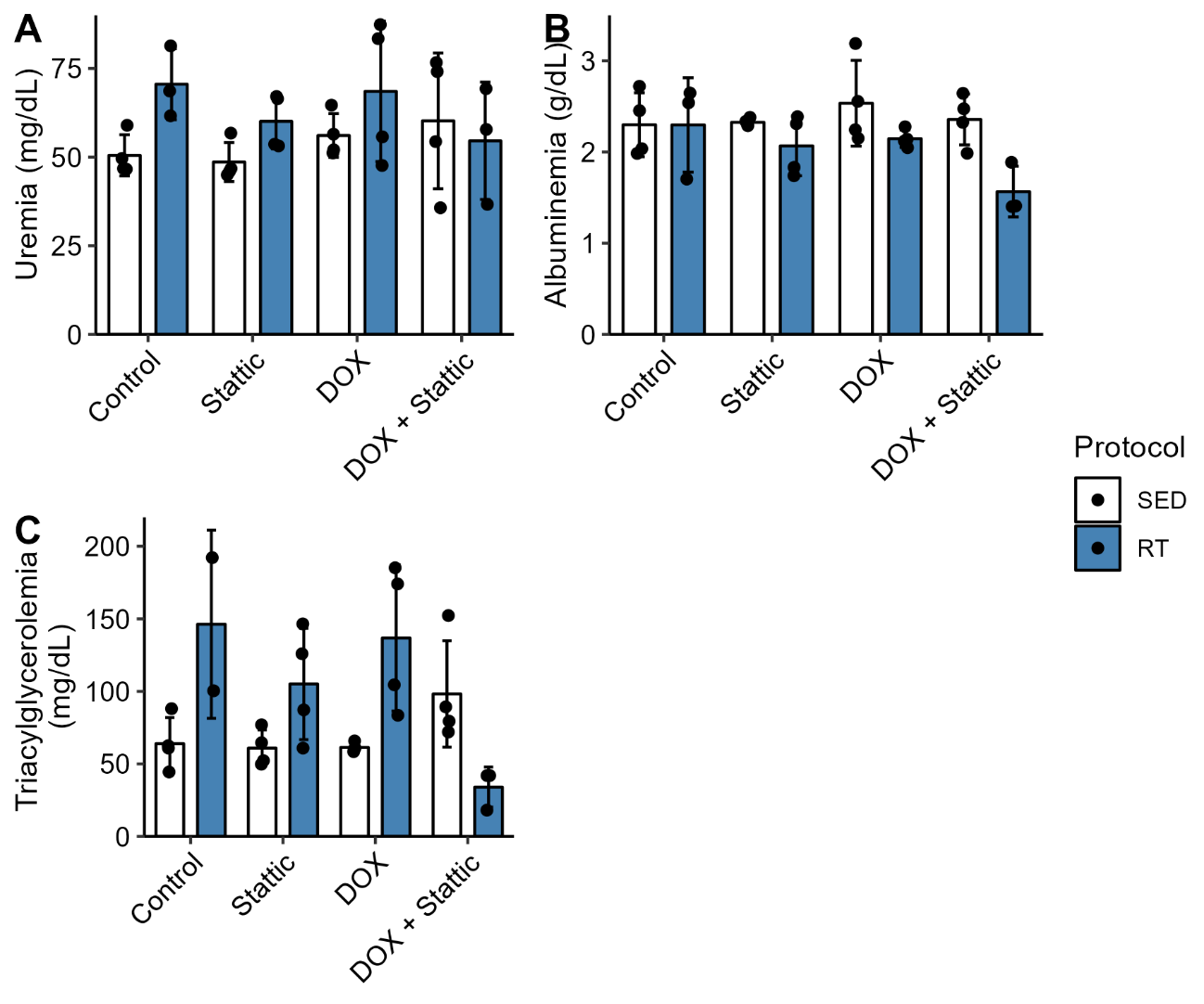


**Figure S1. Serum levels of A) Urea; B) Albumin; C) Triacylglycerol.**

Biochemical serum analysis of uremia, albuminemia and triacylglycerolemia of all experimental groups. Dots represents each individual tested.


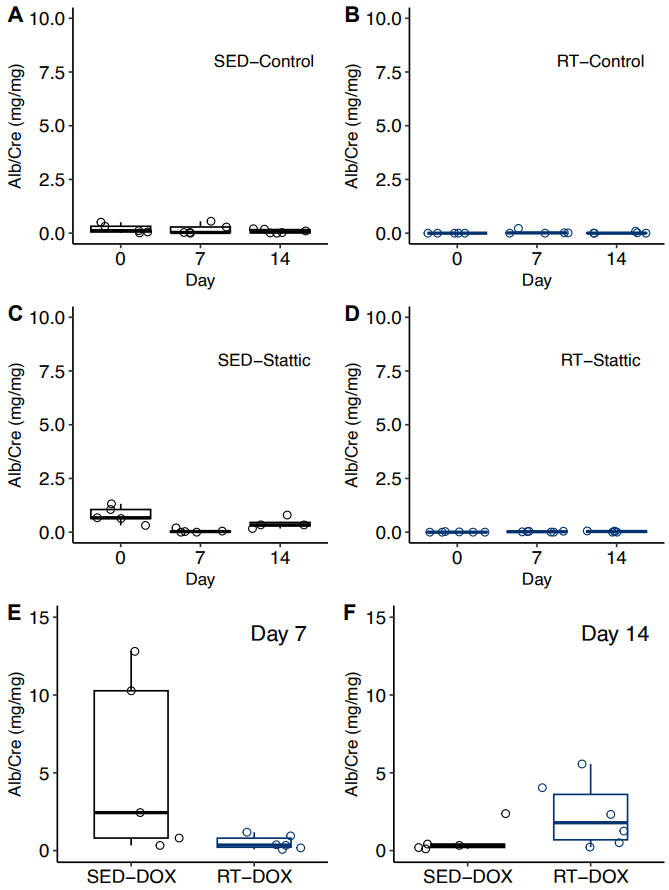


**Figure S2. Analysis of Urinary albumin/creatinine ratio**. **(A-D)** The urinary albumin/creatinine ratio from Control and Stattic groups from both protocols over time (Friedmann test). **(E-F)** Comparison of the albumin/creatinine ratio of DOX at 7 and 14 days in SED and RT protocols (Mann-Whitney test). N = 5 to 7 mice per group.


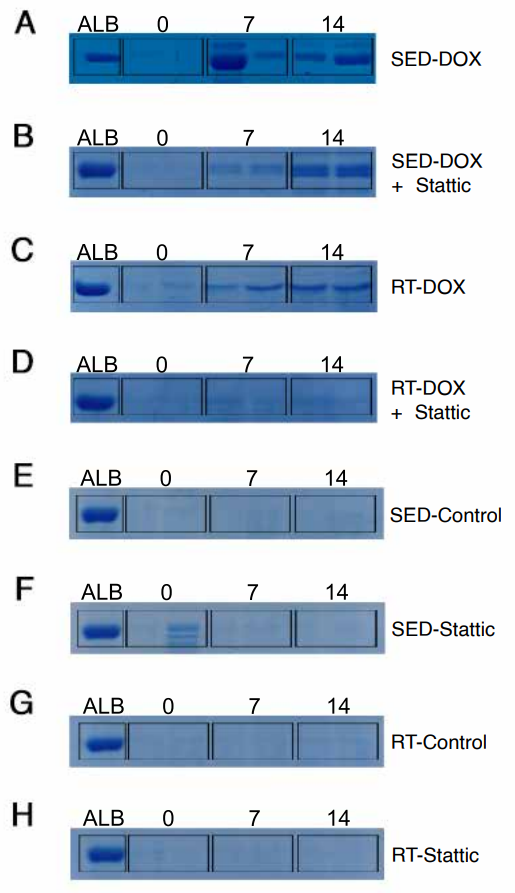


**Figure S3.** Representative acrylamide gels for each experimental group at days 0, 7 and 14 of drugs administration.


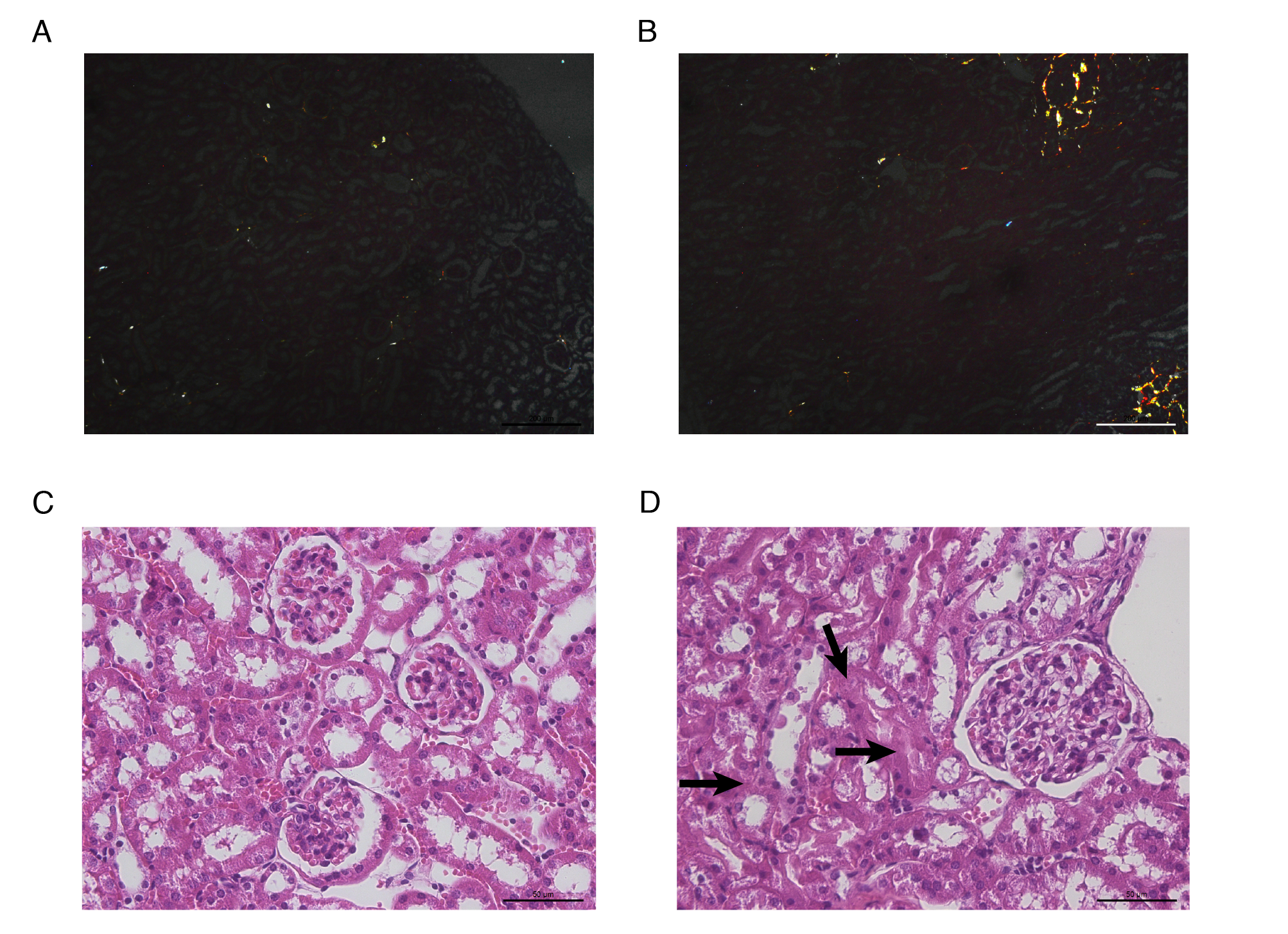


**Figure S4. Representative histological images of picrosirius and H&E staining of kidney tissue**. (A-B) Picrosirius positive areas of (A) control and (B) DOX animal. Scale = 200 µm. (C-D) H&E staining of kidney slices. Arrows indicate areas of fibrotic tissue. (C) Control mouse and (D) DOX mouse. Scale = 50 µm.


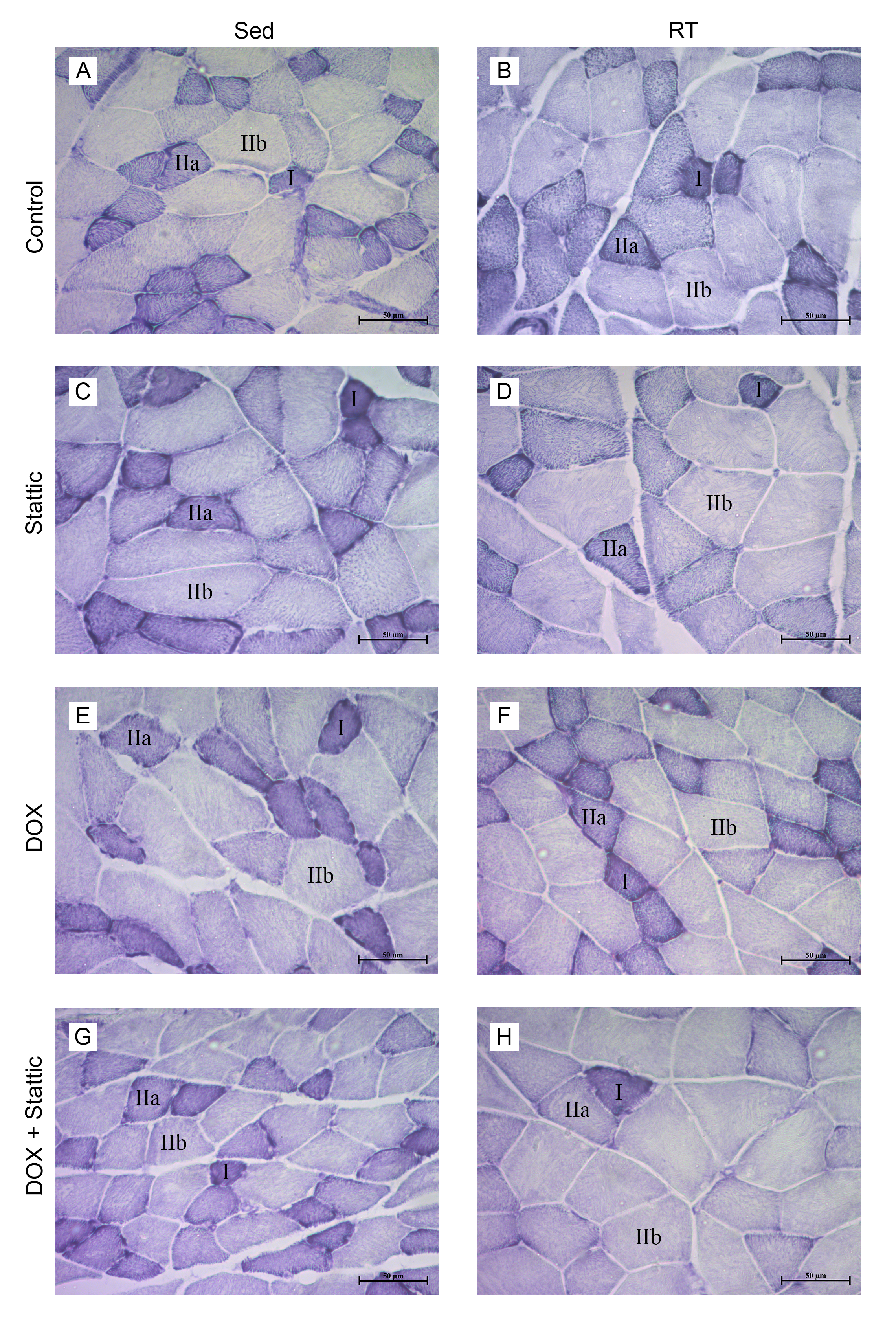


**Figure S5**. **Histological images of muscle fibers in transverse cryosections of gastrocnemius muscle.** Muscle tissues slides at 10 µm were submitted to NADH-TR reaction of fiber typing. I: Type I muscle fiber; IIa: Type IIa muscle fiber; IIb: Type IIb muscle fiber. (A) Sedentary control; (B) Resistance training control; (C) Sedentary Stattic; (D) Resistance training Stattic; (E) Sedentary DOX; (F) Resistance training DOX; (G) Sedentary DOX + Stattic; (H) Resistance training DOX + Stattic. Sed: Sedentary, RT: Resistance training. Scale = 50 µm.


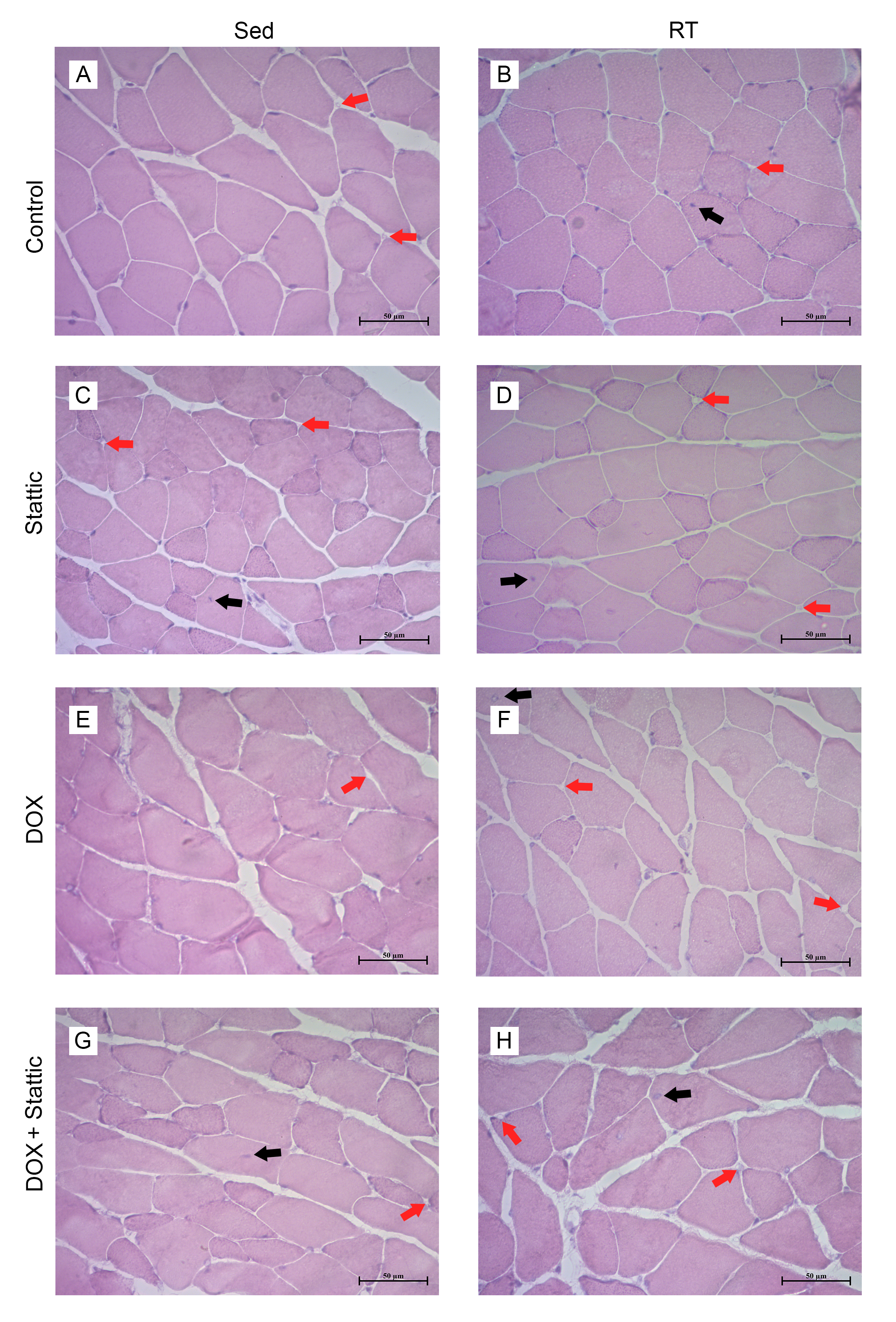


**Figure S6**. **Morphometric characteristics of gastrocnemius muscle fibers stained with hematoxylin and eosin.** Centronucleated myofibers in 10 µm sections are indicate by black arrows; red arrows indicate examples of muscle capillary density. (A) Sedentary control; (B) Resistance training control; (C) Sedentary Stattic; (D) Resistance training Stattic; (E) Sedentary DOX; (F) Resistance training DOX; (G) Sedentary DOX + Stattic; (H) Resistance training DOX + Stattic. Sed: Sedentary, RT: Resistance training. Scale = 50 µm.


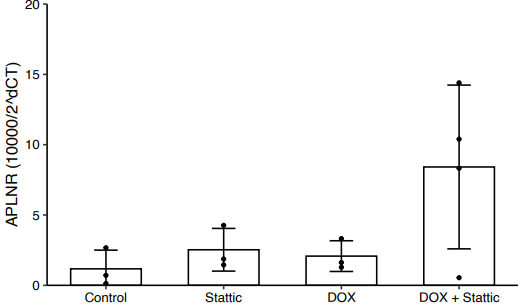


**Figure S7. APNLR levels of sedentary mice.** APLNR expression on gastrocnemius muscle of sedentary mice.


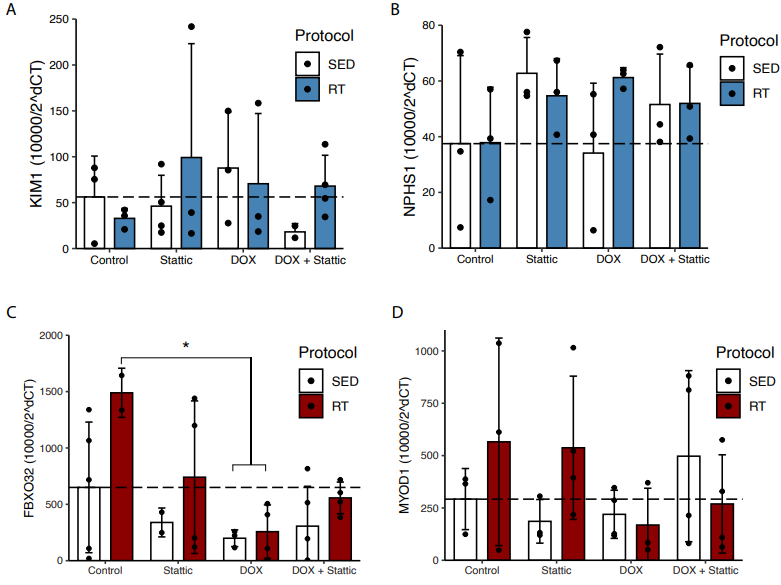


**Figure S8. mRNA levels of KIM-1 and NPHS1 in the kidney and FBXO32 and MYOD1 in the gastrocnemius muscle of mice.** Gene expression of kidney molecules (A-B) and muscle gene expression (C-D).


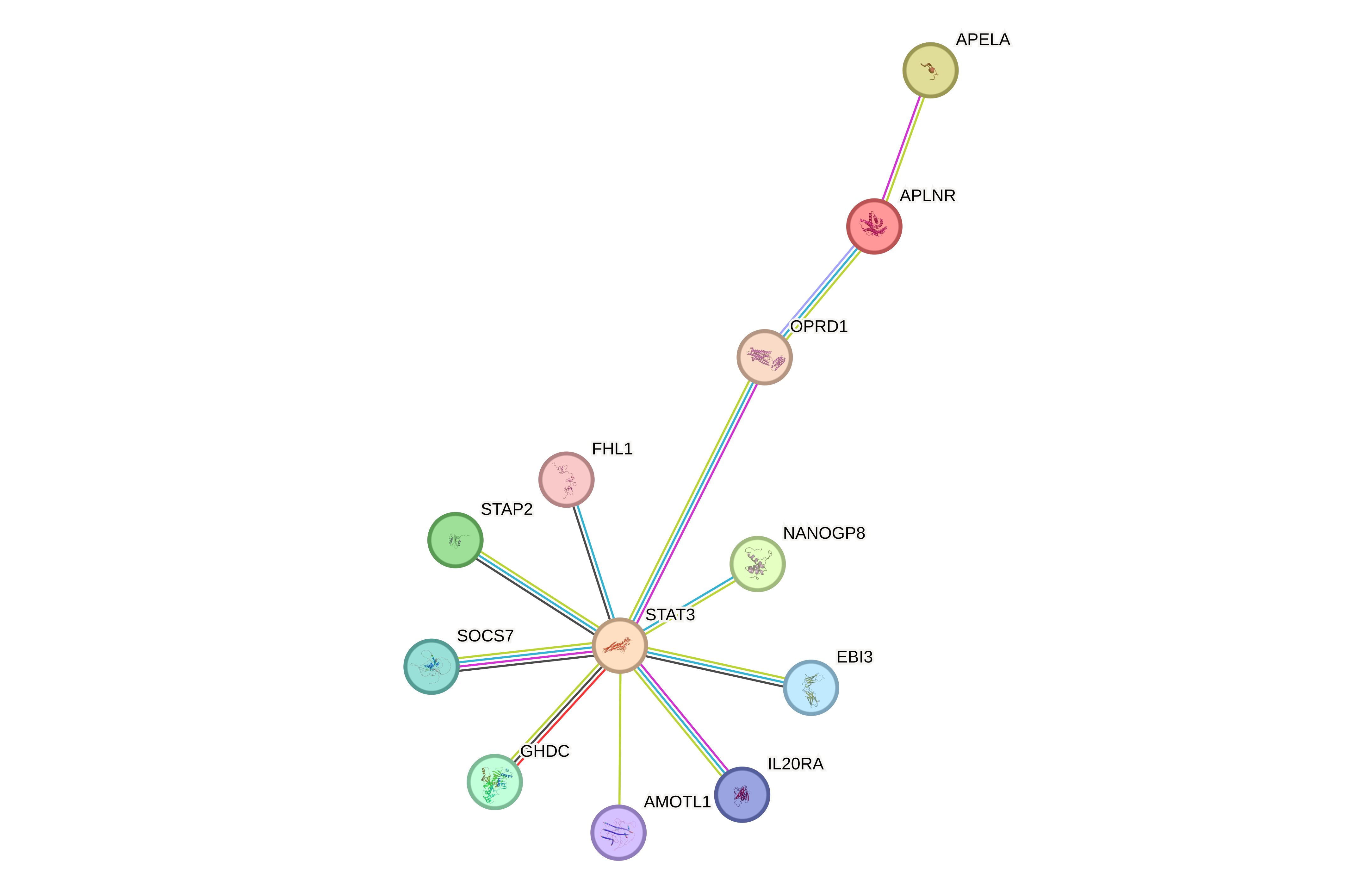


**Figure S9. STRING network of STAT3-APLNR.** Protein-protein interaction network linking STAT3 and APLNR.
